# Hormesis, the Individual and Combined Phytotoxicity of the Components of Glyphosate-Based Formulations on Algal Growth and Photosynthetic Activity

**DOI:** 10.3390/toxics12040257

**Published:** 2024-03-30

**Authors:** Szandra Klátyik, Eszter Takács, Attila Barócsi, Sándor Lenk, László Kocsányi, Béla Darvas, András Székács

**Affiliations:** 1 Agro-Environmental Research Centre, Institute of Environmental Sciences, Hungarian University of Agriculture and Life Sciences, H-2100 Gödöllő, Hungary; klatyik.szandra@uni-mate.hu (S.K.); takacs.eszter84@uni-mate.hu (E.T.); 2 Department of Atomic Physics, Institute of Physics, Budapest University of Technology and Economics, H-1111 Budapest, Hungary; barocsi.attila@ttk.bme.hu (A.B.); lenk.sandor@ttk.bme.hu (S.L.); kocsanyi.laszlo@ttk.bme.hu (L.K.); 3 Hungarian Society of Ecotoxicology, H-1022 Budapest, Hungary; mott@bdarvas.hu

**Keywords:** glyphosate, co-formulants, POEA, APG, algae, phytotoxicity, photosynthetic activity, hormesis, growth inhibition, combined toxicity

## Abstract

The occurrence of the market-leading glyphosate active ingredient in surface waters is a globally observed phenomenon. Although co-formulants in pesticide formulations were considered inactive components from the aspects of the required main biological effect of the pesticide, several studies have proven the high individual toxicity of formulating agents, as well as the enhanced combined toxicity of the active ingredients and other components. Since the majority of active ingredients are present in the form of chemical mixtures in our environment, the possible combined toxicity between active ingredients and co-formulants is particularly important. To assess the individual and combined phytotoxicity of the components, glyphosate was tested in the form of pure active ingredient (glyphosate isopropylammonium salt) and herbicide formulations (Roundup Classic and Medallon Premium) formulated with a mixture of polyethoxylated tallow amines (POEA) or alkyl polyglucosides (APG), respectively. The order of acute toxicity was as follows for Roundup Classic: glyphosate < herbicide formulation < POEA. However, the following order was demonstrated for Medallon Premium: herbicide formulation < glyphosate < APG. Increased photosynthetic activity was detected after the exposure to the formulation (1.5–5.8 mg glyphosate/L and 0.5–2.2 mg POEA/L) and its components individually (glyphosate: 13–27.2 mg/L, POEA: 0.6–4.8 mg/L), which indicates hormetic effects. However, decreased photosynthetic activity was detected at higher concentrations of POEA (19.2 mg/L) and Roundup Classic (11.6–50.6 mg glyphosate/L). Differences were demonstrated in the sensitivity of the selected algae species and, in addition to the individual and combined toxicity of the components presented in the glyphosate-based herbicides. Both of the observed inhibitory and stimulating effects can adversely affect the aquatic ecosystems and water quality of surface waters.

## 1. Introduction

The majority of various pesticide formulations have significant direct or indirect detrimental effects on the environment, particularly in surface waters due to their leaching, surface run-off from treated areas, drifting, foliar spray, and unintended overspray [1,2,3]. Non-selective glyphosate-based herbicides (GBHs) are no exemption from this trend [4,5]. Originally, these herbicides were exclusively applied for pre-emergence weed control. However, the introduction of glyphosate-tolerant genetically modified crops (not authorized for cultivation in the European Union) and the adoption of pre-harvest desiccation practices in agriculture resulted in a substantial increase in the use of glyphosate-based formulations [6,7,8]. However, the approval of the active substance glyphosate has been renewed according to the current legislation subject to the specified conditions and restrictions. Based on the Commission Implementing Regulation (EU) 2023/2660, pre-harvest use of GBHs as desiccants to control the time of harvest or optimize threshing is not authorized [9,10].

Globally, more than 2000 commercial GBHs are used for chemical plant protection against weeds. Different salts of glyphosate (e.g., glyphosate isopropylammonium salt, glyphosate diammonium salt, or glyphosate trimethylsulfonium salt) are used as active ingredients in various GBHs to enhance the solubility of the parent compound, glyphosate [11,12]. In addition to the active ingredient, various co-formulants are also included in GBHs. The primary function of these co-formulants is to facilitate the effectiveness and bioavailability of the formulation by increasing the solubility, adsorption, and absorption of the active ingredient [13]. For example, POEA (a mixture of polyethoxylated tallow amines) as a formulating agent in GBHs enhances the penetration of glyphosate into the plant cell [14]. Various co-formulants presented in commercial pesticides were considered inactive components with regard to the required main biological effect of the formulation. However, numerous studies have indicated the high individual toxicity of co-formulants and the enhanced combined toxicity of the active ingredients and co-formulants in various commercial pesticide formulations compared to the individual toxicity of active ingredients [15,16,17]. Therefore, the use of POEA in GBHs has been banned in the EU due to the incriminating scientific evidence [18].

As a result of excessive global use, glyphosate has become a ubiquitous contaminant in aquatic ecosystems [19,20]. The appearance and concentration of glyphosate in the different environmental elements (e.g., soil, ground and surface waters) are highly influenced by several abiotic (e.g., hydrological conditions, pH, suspended materials), biotic (e.g., activity and composition of the microbial community), and climatic factors (e.g., rainfall frequency and intensity) [21,22,23], in addition to the condition of pesticide treatments (e.g., frequency and timing of the treatment) [22,24]. In the past, glyphosate was not included in standard pesticide monitoring programs. Thus, the environmental concentration of glyphosate and its metabolites were underestimated particularly in regions where pre-harvest desiccation practices are widespread or the cultivation of glyphosate-tolerant genetically modified crops occurs extensively. The primary metabolite of glyphosate, aminomethylphosphonic acid (AMPA), is more mobile in water than the parent compound [25,26] and is frequently detected in various environmental elements, including groundwater and surface waters [26,27,28,29,30,31]. However, it is important to note that the appearance of AMPA in environmental matrices (e.g., groundwater, influents, or sewage sludge) is not exclusively a result of glyphosate metabolism, as it can also originate from phosphonate detergents used in different softeners and cleaning agents [31,32]. According to the U.S. Geological Survey, the presence of glyphosate and/or AMPA was identified in 59% of the analyzed surface waters [33].

The level of glyphosate contamination can reach up to 5.2 mg/L in surface water, although mainly in streams near the treated agricultural fields and especially after heavy rains [27,34,35]. However, high variability can be observed in the detected glyphosate residue levels in various surface water samples [27]. In surface waters collected in Argentina, the average concentrations of glyphosate and AMPA were in the ranges of 17.5–35.2 and 0.6–2.1 µg/L, respectively [36]. However, maximum concentrations were up to 0.258 and 5.87 mg/L, respectively, in the analyzed groundwater and surface water samples [37]. Based on European monitoring programs, the level of glyphosate contamination in surface waters within the EU seems to be relatively lower, with typical glyphosate concentrations ranging between 0.05 and 0.85 µg/L, but residues are consistently detectable [27]. In water samples collected from Hungary, Switzerland, and Italy, the detected glyphosate contamination ranged from 0.035 to 96 µg/L [5,27,35,38,39,40].

Different GBHs manufactured with various co-formulants show different environmental behaviors (e.g., different half-lives and mobility in soil and water). After the pesticide treatments, the active ingredients and the co-formulants rapidly become separated in most cases. The half-life (DT_50_) of glyphosate in water varies from a few to 91 days [41]. Furthermore, the photo- and biodegradation of the active ingredient also occurred in surface waters [41,42], although limited information is available about the half-lives and the environmental fate of the co-formulants [20,43]. In general, most studies focus on the possible toxic effects on various aquatic organisms and the analytical possibilities of the qualitative and quantitative determination of the co-formulants including POEA and APGs. However, the presence of the GBH co-formulant POEA has been observed extensively in soils collected from agricultural fields of the mid-western states in the USA, where the cultivation of genetically modified glyphosate-tolerant crops is concentrated [44]. Additionally, studies have demonstrated the persistence of POEA in soil along with glyphosate and AMPA [44,45,46] and possible access to natural waterways [20,43,45]. Due to their low environmental impacts and biodegradability [47], APGs are commonly used as additives in pesticide formulations, personal skin products, and drugs [48,49]. The environmental concentration of co-formulants is generally not monitored [45]; therefore, the exact concentration of POEA and APGs in surface waters is not known. However, the presence of co-formulants such as POEA and APGs in the environment has been demonstrated (e.g., soil, sediment, wastewater) [43,49]. Typically, glyphosate and the co-formulants presented in the GBHs coexist in environmental matrices (e.g., soil and waters) and such co-exposure can affect various non-target aquatic organisms. The aquatic organisms and communities are highly exposed to water pollution [50], as their contact with xenobiotics in water is unavoidable. Recently, the possible combined toxic effects between active ingredients and co-formulants on the environment and non-target organisms are poorly understood.

The toxic effects of glyphosate and its formulations have been studied in numerous aquatic organisms, such as various algae species [51,52], crustaceans (e.g., *Daphnia magna*) [53], mollusks [54], fish [55], and amphibians [56]. Based on the results of ecotoxicological testing performed on a wide range of aquatic plant and animal organisms, the damage to different physiological and behavioral functions was demonstrated [20]. In aquatic ecosystems, algal communities constitute the primary producer level and the majority of biomass, playing a key role in the oxygen cycle of water and the atmosphere. They also have essential roles in aquatic food webs and nutrient transport processes [57,58]. In addition, some species (e.g., *Ankistrodesmus fulcatus*) can participate in the breakdown of organic pollutants and toxic compounds (e.g., tributyltin) [59]. However, the massive proliferation of certain cyanobacterial (e.g., *Anabaena flos-aquae, Microcystis aeruginosa*) [60] and green algae species (e.g., *Pleodorina indica*) can lead to deterioration of the water quality of surface waters [61]. The determination of the effects of herbicides used for chemical plant protection on algal species is crucial for the toxicological assessment of herbicide formulations. Various algal species are widely used for environmental biological monitoring [62] and bioremediation activities [63]. The different effects of glyphosate, its metabolite, co-formulants, and/or commercial herbicide formulations on green algae and cyanobacterial species are summarized in Table 1 partially based on our previous review about aquatic ecotoxicity of glyphosate, its formulations, and co-formulants [20].

Based on the results of ecotoxicological studies, the inhibitory effects of glyphosate and its formulation on various green algal (e.g., *Chlorella vulgaris, Scenedesmus incrassatulus, Pseudokirchneriella subcapitata*) [64,65] and cyanobacterial (e.g., *M. aeruginosa*) species were observed [66]. However, stimulated growth was observed at lower test concentrations after exposure to glyphosate and glyphosate-based formulations [64,65,67].

In addition, altered cell morphology, disrupted ultrastructure (e.g., damaged thylakoids and mitochondria) as well as altered biochemical and physiological parameters (e.g., antioxidant activity, lipid peroxidation) were also demonstrated in algae [52,68,69]. Additionally, differences were observed in the sensitivity of the investigated aquatic organisms, even with similar lifestyles, habitats, or identical taxa [70,71,72]. For example, the determined 72 h EC_50_ values are in the range of 24.7–166 mg/L [15,20,73] for *P. subcapitata*, while for *Desmodesmus subspicatus* higher values were calculated (72.9–166 mg/L) [41,52,74,75,76] during the ecotoxicological testing on the effects of glyphosate. Moreover, potential adverse effects of glyphosate and GBHs were indicated also on freshwater periphyton [77,78,79]. Moreover, the increased toxicity of Roundup was demonstrated on cyanobacterial and green algal species (*M. aeruginosa, Nitella microcarpa var. wrightii*) in the presence of POEA [80]. The 72 h EC_50_ values for POEA in *P. subcapitata* ranged from 0.2 to 4.9 mg/L [15,81,82]. The negligible aquatic toxicity of APGs was demonstrated on *P. subcapitata* [83], but the toxicity of APGs highly depends on the length of the of the carbon chain [84,85].

In addition to growth inhibition, photosynthetic activity is a commonly used endpoint during the assessment of phytotoxic effects. The measurement of photosynthetic parameters ensures a non-invasive and rapid indication of harmful effects. A widely used method for the measurement of photosynthetic activity is the detection of induced chlorophyll-a fluorescence [86]. Recently, the measurement of photosynthetic activity is widely used in research on stress effects on plant organisms, and for characterizing the physiological state of plants [87,88,89]. In addition to herbicide active ingredients that directly inhibit photosynthesis (e.g., atrazine), additional active ingredients, including glyphosate, can also impact photosynthetic and respiratory processes by influencing various metabolic pathways [90,91]. The adverse effects of glyphosate on photosynthetic processes can be explained by the direct or indirect inhibition of plastoquinone biosynthesis [92,93]. Furthermore, the reduction in chlorophyll concentration [94] directly affects the rate of electron transport in the chloroplast [91]. Reactive oxygen species generated in mitochondria can further affect photosynthesis by inhibiting the respiratory electron transport chain as a result of glyphosate exposure. The generated free radicals leave mitochondria and enter the chloroplast, where they cause oxidative damage to the photosynthetic apparatus and reduce the activity of photosynthesis [94]. The phytotoxic effects of glyphosate and its herbicide formulation on photosynthetic activity have been studied on phytoplankton species [95,96]. The observed effects indicated damage to the photochemical efficiency of the PS II photochemical system [95]. However, increased growth, chlorophyll-a content, and photosynthetic activity were observed at lower concentrations [90].

**Table 1:** Effects of glyphosate, its metabolite, co-formulants, and/or commercial herbicide formulations on green algae and cyanobacterial species.

Algae Species	Tested Substances	Test Concentrations	Test Period	Tested Parameters	Main Results	Reference
*P. subcapitata*	technical-grade glyphosate (GLY) acid, GLY-IPA ^a^, Roundup, POEA ^b^	dilution series	96 h ^c^	growth inhibition	96 h IC_50_^d^ = 3.92 mg a.e. ^e^ /L (POEA), 5.81 mg a.e./L (Roundup), 24.7 mg a.e./L (GLY acid), 41.0 mg a.e./L (GLY-IPA)	[15]
*P. subcapitata*	Roundup	4.7–60 mg/L	96 h	growth inhibition	96 h EC_50_^f^ = 15.60 mg/L, damaged cell ultrastructure	[52]
*C. vulgaris*	GLY, AMPA ^g^	0.05–50 mg/L, individual and co-exposures	7 d ^h^	growth inhibition, pigment content, antioxidant activity	stimulated growth (≤ 0.5 mg/L), growth inhibition (≥ 5 mg/L), inhibitory effect (≥ 5 mg/L GLY and AMPA), altered pigment levels, increased antioxidant activity	[64]
cyanobacteria, *Chlorophycean* microalgae	GBH ^i^ (Faena)	1–100 mg/L	96 h	growth inhibition, antioxidant enzymes	IC_50_ = 1.022–2.702 mg/L, affected antioxidant enzyme activity (≥ 0.74 mg/L)	[65]
*M. aeruginosa*	GLY	1–10 mg/L	9 d, enzyme assays: 24–48 h	growth inhibition, chl-a ^j^ content, antioxidant activity, cell apoptosis	reduced growth and chl-a content, increased antioxidant activity (1–2 mg/L), induced apoptosis	[66]
cyanobacterial strains	GLY	8.5–33.8 mg/L	15 d	growth inhibition, phosphate and phosphonate levels	species- and dose-dependent stimulatory effects, decreased phosphonate levels, concentration-dependent phosphate uptake	[67]
*S. vacuolatus*	GBH (Glifosato Atanor) with 2.5% of the surfactant (alkyl aryl polyglycol ether)	0–8 mg GLY/l	96 h	growth, morphology, oxidative stress parameters	96 h IC_50_ = 4.9 mg/L, metabolic and morphological changes (≥ 4 mg/L), oxidative damage (≥ 6 mg/L)	[68]
cyanobacterial species	pesticide adjuvants	dilution series	96 h	growth inhibition	substance- and species-specific effects	[71]
*N. microcarpa* var. *wrightii*	technical-grade GLY, GBH (Roundup), AMPA	GLY, Roundup: 0.28, 3.5, 6 mg/L; AMPA: 0.03 mg/L	7 d	photosynthetic rate, dark respiration rate, chl-a	higher toxicity of Roundup, stimulatory effect of AMPA	[80]
*P. subcapitata*	POEA	dilution series	96 h	growth inhibition	96 h EC_50_ = 4.1–4.9 mg/L	[81]
*P. subcapitata* *C. vulgaris*, *Oophila sp*	MON 0818	dilution series	96 h	growth inhibition	96 h EC_50_ = 0.21–1.61 mg/L	[82]
*P. subcapitata*	APG ^k^	dilution series	72 h	growth inhibition	negligible aquatic toxicity	[83]
*P. subcapitata*	APG	dilution series	72 h	growth inhibition	toxicity affected by the length of the carbon chain	[84]
green microalgae species	APG	0.26–6.8 mg/L	72 h	growth inhibition	72 h EC_50_ = 0.32–2.7 mg/L	[85]
*M. aeruginosa*	GLY, Roundup	0.06–29.6 µg/L	21 d	cell number, chl-a, APA ^l^ activity	increased cell number and chl-a, inhibition (> 5.92 µg/L), GLY increased photosynthesis, concentration-dependent APA activity	[90]
freshwater microalgae	GLY	maximum tested concentration: 5.07 g/L	80 min	chl-a fluorescence, cell viability	concentration-specific effect on maximum quantum yield of PSII ^m^ (< 0.17 mg/L)	[95]
microalgal and cyanobacterial species	Factor 540R	10–1000 µg/L	48 h	growth inhibition, photosynthetic parameters	48 h EC_50_ = 406–724 µg/L, modified photosynthetic response (≥ 10 µg/L)	[96]

^a^ glyphosate isopropylammonium salt; ^b^ mixture of polyethoxylated tallow amines; ^c^ hour; ^d^ half-maximal inhibitory concentration; ^e^ acid equivalent; ^f^ 50% effective concentration; ^g^ aminomethylphosphonic acid; ^h^ day; ^i^ GBH: glyphosate-based herbicide; ^j^ chlorophyll-a; ^k^ alkyl polyglycoside; ^l^ alkaline phosphatase activity; ^m^ photosystem II.

The aim of this study was to assess the individual and combined acute phytotoxicity of the components of glyphosate-based formulations. During the comparison of toxic effects, glyphosate was tested in the form of pure active ingredient (glyphosate isopropylammonium (IPA) salt) and preparations (Roundup Classic and Medallon Premium) formulated with a mixture of polyethoxylated tallow amines (POEA) or alkyl polyglucosides (APG), respectively. In addition, the individual toxicity of the formulating agents (POEA and APG) was also investigated. During our study, standard algal growth inhibition assays were performed on different green algae species (*D. subspicatus*, *P. subcapitata*, *Scenedesmus obtusiusculus*) and a cyanobacteria (*A. flos-aquae*). Based on the results, the differences in the sensitivity of various algal species were also compared. In addition, we investigated the possible effects on the photosynthetic activity of *P. subcapitata* algae cells exposed to the components of Roundup Classic individually and in combination.

## 2. Materials and Methods

### 2.1. Standard and Reagents

Glyphosate IPA salt and the mixture of polyethoxylated tallow amines (POEA, under the tradename: Emulson AG GPE 3SS) were received from Lamberti SpA (Albizzate, Italy). The glyphosate-based Roundup Classic (Monsanto Europe S.A./N.V.) [97] and Medallon Premium (Syngenta) [98], in addition to the alkyl polyglucosides (APG, under the tradename: Plantapon LGC) were purchased from a public commercial source. The main chemical properties of the investigated herbicide active ingredient, formulations, and surfactants (POEA and APG) used in the investigated formulations can be found in Table 2. Based on the Material Safety Data Sheet (MSDS), Roundup Classic contains 41.5% glyphosate IPA salt and 15.5% POEA. In addition, Medallon Premium consists of 34% glyphosate diammonium salt and 10–20% APG. However, the selected formulations contain different salts of glyphosate, and the indicated concentrations of the active ingredient correspond to 360 g/L glyphosate acid concentration for both preparations. During the ecotoxicological testing, glyphosate was tested only in the form of glyphosate IPA salt. In the tested concentration ranges, the water solubility is not limited for any forms of glyphosate active ingredient, and in water, the salts of glyphosate quickly dissociate into ions that are also found in nutrient solutions and buffer solutions used in ecotoxicological studies.

### 2.2. Selected Algae Monocultures

The selected algae species were obtained from public collections. The green algae *Pseudokirchneriella subcapitata*, Korshikov (NIVA-CHL1, previous name: *Selenastrum capricornutum*, current name: *Raphidocelis subcapitata*) was obtained from the alga collection of the Norwegian Institute for Water Research. The additional *Desmodesmus subspicatus*, Hegewald & Schmidt (CCAP 276/20) and *Scenedesmus bijugus var. obtusiusculus*, Schmidt (CCAP 276/25) green algae species, as well as the investigated filamentous cyanobacteria *Anabaena flos-aquae* (CCAP 1403/13D, current name: *Dolichospermum flos-aquae*), were derived from the Scottish Culture Collection of Algae and Protozoa. The batch culture of green algae species and the selected cyanobacteria were maintained in Zehnder-8 (pH = 6–7) [99] and Allen (pH = 6–7) [100] media, respectively. Fresh media were added to the algae cultures every two weeks, and they were maintained at 20 ± 2 °C and illuminated in a 14:10 light/dark period with the use of cool-white fluorescence tubes (15 µmol/m^2^/s). The sensitivity of the algae cultures was verified with the use of the reference substance (potassium dichromate, K_2_Cr_2_O_7_) before testing and was proven to be acceptable (72 h EC_50_ = 1.0 ± 0.1 mg/L) within the appropriate ranges (0.8 ± 0.1 mg/L for *D. subspicatus*; 1.2 ± 0.3 mg/L for *P. subcapitata*) based on the relevant standard protocol [101].

The selected green algal and cyanobacterial strains are sensitive to changes in water quality, so they serve as excellent test organisms for the investigation of the toxic effects of aquatic pollutants. *P. subcapitata* and *D. subspicatus* are also considered reference species recommended by the related OECD guideline [102]. Additionally, the selected strains can be easily maintained under laboratory conditions, and are characterized by a fast reproduction and life cycle [103,104]. Based on the scientific literature, significant differences can be observed in the sensitivity of algal species to certain pollutants even within the same taxa [71]. To investigate and compare the sensitivity of taxonomically close and distant species to the applied treatments, three common representatives of freshwater green algae (Phylum: *Chlorophyta*) were selected. Two of the selected green algae species (*D. subspicatus* and *S. obtusiusculus*) belong to the same taxonomic family (*Scenedesmaceae*), thus the sensitivity can be compared also in the case of taxonomically very close species. With the use of *A. flos-aquae* representing a species of cyanobacteria known for its ability to form harmful algal blooms [60], the differences in the sensitivity to the effects of glyphosate can be evaluated for eukaryotic green algae cells and a prokaryotic cyanobacterium as well.

### 2.3. Algal Growth Inhibition Tests

The individual and combined phytotoxic effects of the components of the tested GBHs were evaluated in algal growth inhibition tests based on the OECD 201 guideline [102]. Growth inhibition tests were performed on three unicellular green algae species (*P. subcapitata*, *D. subspicatus*, *S. obtusiusculus*) and, in the case of the active ingredient glyphosate, the tests were also performed on the selected filamentous cyanobacteria (*A. flos-aquae*). The duration of the test was 72 h. During the tests, continuous and uniform cool-white illumination (104.9–14.9 µE/m^2^/s), optimal pH of algal media (pH = 6–7 for Zehnder-8 and Allen media, as well), controlled temperature (22 ± 2 °C) and stirring (continuous, 100 rpm) were ensured in a shaking incubator (Witeg WIS-10RL, Wertheim, Germany) [102]. The tested compounds were serially diluted, and five concentrations of the substance along with the control were investigated in three repetitions at each level. Each test was repeated three times for each investigated compound. The initial number of algae cells was 10^5^ cells/mL in the tested and control groups with the fulfillment of the conditions for exponential growth during the entire exposure time. During the growth inhibition assays, the algal cell density was determined daily in the control group to monitor the required specific reproduction rate. The concentration ranges used in the algal growth inhibition tests were as follows for the different species: (1) *P. subcapitata*: glyphosate IPA salt: 22–352 mg/L, Roundup Classic: 3.5–56 mg/L, Medallon Premium: 45–720 mg/L, POEA: 0.5–8 mg/L, APG: 6.5–104 mg/L; (2) *D. subspicatus*: glyphosate IPA salt: 22–352 mg/L, Roundup Classic: 7–112 mg/L, Medallon Premium: 95–1520 mg/L, POEA: 0.8–13 mg/L, APG: 10–160 mg/L; (3) *S. obtusiusculus*: glyphosate IPA salt: 22–352 mg/L, Roundup Classic: 15–240 mg/L, Medallon Premium: 125–2000 mg/L, POEA: 1.5–24 mg/L, APG: 30–480 mg/L; (4) *A. flos-aquae*: glyphosate IPA salt: 4.5–36 mg/L.

At the end of the experiments, we determined the amount of algal biomass in each control and treated group. Algal biomass was characterized by the measurement of optical density and chlorophyll-a content. The optical density of green algae cells was determined at a wavelength of 750 nm using a spectrophotometer (UV/VIS Camspec single beam M330, Camspec, Crawley, UK), also in three repetitions for each sample [101]. In addition to the measurement of optical density, the potential toxic effects were also evaluated based on the chlorophyll-a content of the samples in the tests performed on green algae species exposed to the POEA-formulated herbicide and its components. Due to the filamentous structure of *A. flos-aquae*, more reliable results were obtained with the measurement of the chlorophyll-a content. The correlation between the two test methods proved to be very high in the case of green algae species (R^2^ > 0.998). After the extraction process, the chlorophyll-a content of the samples was also determined using a spectrophotometric method in the three replicates [105]. In the performed tests, the coefficient of variation for section-by-section specific growth rates (days 0–1, 1–2, and 2–3) remained below 35% in the control groups. During the entire duration of the tests, the coefficient of variation of the specific growth rates did not exceed 7% in the parallel control cultures of *P. subcapitata* and *D. subspicatus*. In addition, more than 16-fold growth was detected in the control groups, thus the tests can be considered valid.

During the testing of individual and combined effects on algal growth, algae cells were exposed to glyphosate IPA salt and the tested surfactants (POEA and APG) individually and in the form of formulated herbicides. The individual and combined toxicity of the tested substances was evaluated by the determined 72 h EC_50_ values. The 72 h EC_50_ values were calculated based on the measured optical densities and chlorophyll-a contents as well. During the comparative study of the individual and combined toxicity, the 72 h EC_50_ values determined for the tested GBH formulations were corrected with the nominal content of the active ingredient glyphosate and the surfactant as well, based on the MSDS (Table 2).

### 2.4. Photosynthetic Activity Tests

The individual and combined effects of the components of Roundup Classic were assessed on the photosynthetic activity of *P. subcapitata* green algae. The photosynthetic activity was determined in the samples derived from the algal growth inhibition tests after the 72 h exposure. The measurements were carried out with a portable FluoroMeter Module (FMM) device based on the detection of laser-induced chlorophyll-a fluorescence [106]. The measuring principle of the instrument is based on the “Kautsky effect” [86]. Under dark conditions, the photochemical process of photosynthesis in plant cells temporarily ceases, and upon sudden high-intensity stimulation, typically by laser excitation, the chlorophyll molecules in the cells immediately begin to absorb light. However, the optimal conditions for photosynthesis develop more slowly, so only a small fraction of the energy of the light absorbed at the beginning is used in the process of photosynthesis. The excess light energy is re-emitted by the cells in the form of fluorescent light. After a few minutes, as the photosynthetic process resumes, the plant cell utilizes the absorbed light with higher efficiency, causing the intensity of fluorescent radiation to gradually decrease, stabilizing at a lower value [86,107].

During the 96-well microplate-based assay, the photosynthetic parameters were measured after a 10 min dark adaptation with the use of a special sample holder. After the dark acclimatization, the samples were excited with a laser diode (10 mW) at the wavelength of 635 nm. After excitation, the duration of the measurement was 5 min. During the measurements, the intensity of the fluorescent light emitted by the sample was detected at wavelengths of 690 nm and 735 nm [106,107]. The measurements were performed in triplicates. Photosynthetic activity of the control and treated groups was characterized by the observed ratio of fluorescence decrease (Rfd*) and the proxy of quantum efficiency of the algae photosystem PSII (Fv*/Fp). Here, Fp means the peak fluorescence value derived from the fluorescence induction curve using the FMM module, while Fv* represents the variable fluorescence in terms of Fp. Essentially, the Fv/Fm parameter describes the impact of plant stress on photosystem II in a dark-acclimated state, where Fm is the maximum chlorophyll fluorescence under a saturating radiation pulse in such conditions [108,109]. As the maximum actinic level available with the FMM will not saturate PSII, Fp is used to distinguish it from Fm, which represents the maximum fluorescence value during continuous excitation under full saturation [88,108]. Rfd* corresponds to Fd/Fs, where Fs is the observed steady-state fluorescence and Fd indicates the fluorescence reduction from Fp to Fs [106,108]. Detailed explanations of different fluorescence parameters are summarized in Table 3. The effects on photosynthetic activity were compared based on the values detected at the wavelength of 690 nm [88].

### 2.5. Statistical Analysis

Based on the results of algal growth inhibition tests, 72 h EC_50_ values for both measured parameters (optical density and chlorophyll-a content) were determined using the ToxRat Pro 3.0 statistical software (ToxRat Solutions Gmbh, Alsdorf, Germany). The additional statistical analyses were performed with the use of the R Statistical program 4.2.1. (R Development Core Team, Vienna, Austria). The effects of the individual and combined exposures, in addition to the differences between the determined 72 h EC_50_ values, and the detected parameters of photosynthetic activity (Fv*/Fp and Rfd*) were evaluated with the use of general linear models. Before the statistical analysis, the normality of the data and the homogeneity of variance were checked by Shapiro–Wilk and Levene’s or Bartlett’s tests at the significance level of 0.050. Furthermore, the applicability of the fitted model was verified in each case with diagnostic plots (residual variances, QQ plot, Cook’s distance plot). Tukey’s honest significant difference (HSD) tests were used as post hoc analyses to assess the significant differences between groups. The data were evaluated using the Kruskal–Wallis test, if the conditions for applying the chosen model were not met, with the use of the Student–Newman–Keuls (SNK) test for the comparison of the different groups at the significance level of 0.050. In addition, the observed hormetic effects were verified with the use of Brain–Cousens hormesis models available in the ’dcr’ package of the R Statistical program 4.2.1. [110,111,112].

## 3. Results

### 3.1. Individual and Combined Effects on Algal Growth

During the ecotoxicological testing, significant differences were not observed in the 72 h EC_50_ values determined for the active ingredient glyphosate based on the optical density of *P. subcapitata* (125.2 ± 16.5 mg/L) and *D. subspicatus* (132.9 ± 2.3 mg/L) green algae samples (*p* = 0.467). On the other hand, much higher individual toxicity of glyphosate was demonstrated on *S. obtusiusculus* (73.1 ± 21.2 mg/L) compared to the individual toxicity values determined for the two other green algae species (*p* < 0.001). The individual toxicity of POEA significantly exceeded the individual toxicity of glyphosate for all three algal species (*p* < 0.001). Furthermore, compared to the individual toxicity of glyphosate, the toxicity of the formulation significantly increased in the presence of POEA during the examination of all species (*P. subcapitata*, *D. subspicatus*: (*p* < 0.001); *S. obtusiusculus p* = 0.005). In the case of the formulation, no difference can be observed between the toxicity values corrected with the nominal content of POEA and determined after the individual exposure to POEA for *P. subcapitata* (*p* = 0.146) and *D. subspicatus* (*p* = 0.172), but the individual toxicity of POEA was lower on *S. obtusiusculus* (*p* = 0.021) (Table 4). Based on the determined 72 h EC_50_ values, a significant difference can be observed in the sensitivity of the tested species. There is no difference between the sensitivity of *P. subcapitata* and *D. subspicatus* (*p* = 0.467) for glyphosate, while the sensitivity of *S. obtusiusculus* was higher due to the toxic effects of glyphosate (*p* < 0.001). In contrast, *S. obtusiusculus* was the most tolerant against the effects of the POEA-formulated herbicide and POEA, followed by *D. subspicatus*, while in the case of both tested substances, *P. subcapitata* proved to be the most sensitive green algae (Roundup Classic: *p* < 0.001; POEA: *p* < 0.030) (Table 4).

Similar to the toxicity values based on the optical density measurements, the toxicity of the POEA-formulated herbicide was also higher compared to the individual toxicity of glyphosate on the investigated green algae species (*p* < 0.001), according to the 72 h EC_50_ values based on the measurements of the chlorophyll-a content. The highest individual toxicity of glyphosate (17.4 ± 6.0 mg/L) was demonstrated for the tested cyanobacterium (*A. flos-aquae*) (*p* < 0.001). However, based on the chlorophyll-a content, a difference can be observed in the sensitivity of the two green algae, as *D. subspicatus* proved to be more sensitive (73.8 ± 5.3 mg/L) compared to *P. subcapitata* (105.3 ± 17.8 mg/L) (*p* = 0.004). The individual toxicity of POEA also proved to be much higher compared to the individual toxicity of glyphosate based on the chlorophyll-a content (*p* < 0.001), where *P. subcapitata* was more sensitive to the effect of POEA (*p* < 0.001). In contrast to the active ingredient, there was no significant difference between the 72 h EC_50_ values for the formulation corrected with the POEA content and the determined toxicity values for POEA alone on the tested green algae species (*p* ≥ 0.096). Moreover, differences were not indicated in the sensitivity of the green algae species exposed to the formulation (*p* = 0.838) (Table 5).

According to the results of algal growth inhibition tests performed on the APG-formulated herbicide, differences were not demonstrated between the individual toxicity of glyphosate and the combined toxicity indicated by the calculated 72 h EC_50_ values for the formulation on *P. subcapitata* (*p* = 0.856). In contrast to the POEA-formulated GBH, the individual toxicity of glyphosate significantly exceeded the combined toxicity of the components determined in the form of the APG-formulated herbicide on the other two tested green algae species (*D. subspicatus*: *p* < 0.001, *S. obtusiusculus*: *p* = 0.002). *S. obtusiusculus* proved to be more sensitive to the effects of glyphosate (*p* = 0.001) (Table 6). Similar to POEA, the individual toxicity of APG was higher compared to the individual toxicity of glyphosate on *P. subcapitata and D. subspicatus* (*p* < 0.001), where *P. subcapitata* proved to be more sensitive (*p* < 0.001). Conversely, the individual toxicity of glyphosate was higher compared to the individual effects of APG on *S. obtusiusculus* (*p* = 0.008). Significant differences were not detected between the toxicity values determined for Medallon Premium corrected with the APG content and indicated after the individual exposure to the surfactant APG on *P. subcapitata* (*p* = 0.068) and *S. obtusiusculus* (*p* = 0.109), similarly to POEA. However, the individual toxicity of APG was higher compared to the combined effects of the components indicated by toxicity values for the APG-formulated herbicide corrected with the APG content on *D. subspicatus* (*p* = 0.001). Based on the determined 72 h EC_50_ values, significant differences can be observed in the sensitivity of the tested species. *P. subcapitata* was the most sensitive species for both the formulation and APG (Medallon Premium: *p* < 0.001, APG: *p* ≤ 0.001). In the case of the additional two green algae, the difference was not demonstrated in their sensitivity to the effects of the APG-formulated herbicide (*p* = 0.650), while *S. obtusiusculus* proved to be more tolerant to the toxic effects of APG (*p* < 0.001) (Table 6).

### 3.2. Effects on the Photosynthetic Activity of Green Algae Cells

**Figure 1:**
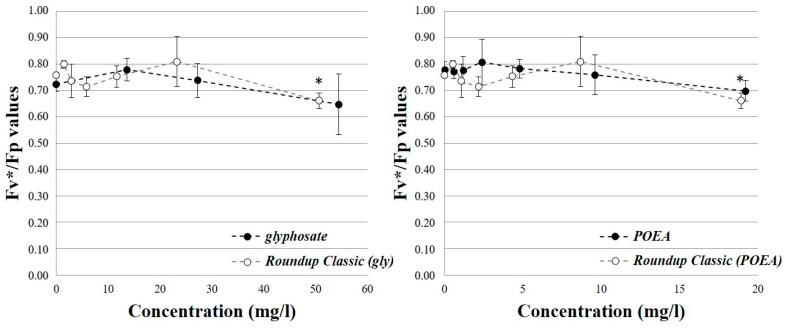
The individual and combined effects of glyphosate and POEA on the Fv*/Fp values characterizing the photochemical efficiency of the PS II photochemical system in the exposed algae cells.

The individual and combined effects of the components presented in the tested POEA-formulated herbicide on the photosynthetic activity of *P. subcapitata* were evaluated according to the measured Fv*/Fp values connected to the photochemical efficiency of the PS II photochemical system and Rfd* values characterizing photosynthetic activity. During the investigation of the effects of glyphosate on photosynthetic activity, the pure active ingredient did not result in a significant decrease in the Fv*/Fp values compared to the control group up to a concentration of 109 mg/L (*p* = 0.034). In contrast to the pure active ingredient, the formulation resulted in a significant reduction in the Fv*/Fp value (*p* = 0.015) compared to the control group, but only at the highest tested concentration (50.6 mg/L). After the individual exposure to POEA, significant changes in Fv*/Fp values were not observed in the tested concentration range (0.6–19.2 mg/L). POEA in the presence of the active ingredient did not cause the reduction in the Fv*/Fp value up to the highest tested concentration (18.9 mg/L) compared to the control group (*p* < 0.001) (Figure 1—the Fv*/Fp values were plotted in the common concentration range of the tested components: glyphosate: 0–54.5 mg/L; POEA: 0–19.2 mg/L).

**Figure 2:**
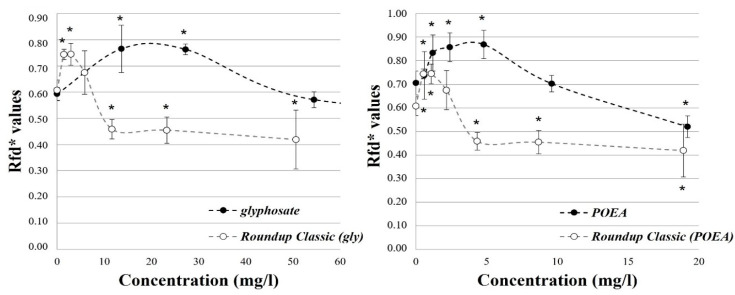
The individual and combined effects of glyphosate and POEA on the Rfd* values characterizing photosynthetic activity in the exposed algae cells.

During the investigation of glyphosate, a significant increase was observed in the Rfd* values at the lower tested concentrations (13.6–27.2 mg/L) (*p* < 0.025). However, above this range, no significant difference was observed compared to the control group, not even at the highest concentration (436 mg/L) (*p* = 1.000). After the exposure to glyphosate in the form of herbicide formulation, increased Rfd* values were also detected at the lower test concentrations (1.5–5.8 mg/L) compared to the control group. However, the difference was significant only at the two lowest concentrations (*p* < 0.018). In contrast, significantly decreased Rfd* values were observed at the higher concentration range (11.6–50.6 mg/L) (*p* < 0.012) (Figure 2—the Rfd* values were plotted in the common concentration range of the tested components: glyphosate: 0–54.5 mg/L; POEA: 0–19.2 mg/L). Similar to the effects of glyphosate, a significant increase in Rfd* was observed after the individual exposure to POEA at the lower concentration range (0.6–4.8 mg/L) compared to the control group (*p* < 0.035). However, a significant decrease was demonstrated in the Rfd* values (*p* = 0.009) at the highest tested concentration (19.2 mg/L). After the exposure to the herbicide formulation, POEA also resulted in an increase in Rfd* values at the lower concentration range of POEA (0.5–2.2 mg/L) compared to the control. However, significant differences were not observed at the higher concentrations (4.8–19.2 mg/L) (*p* > 0.984) (Figure 2).

## 4. Discussion

According to the determined 72 h EC_50_ values based on the measured optical density and chlorophyll-a content of the samples, the results and observed trends in toxicity correlated well between the tested endpoints. However, higher differences can be observed in some cases. Generally, lower toxicity values were determined based on the chlorophyll-a content compared to the 72 h EC_50_ values calculated based on the optical density of the samples (Table 4 and Table 5). The observed differences between the 72 h EC_50_ values based on the two tested endpoints can presumably be explained by the fact that the determination of the optical density can be disturbed by the aggregation of cells and the presence of the remains of dead cells in the sample. On the other hand, during the analytical determination of the chlorophyll-a content, this disturbing matrix is not presented after the extraction of the samples. The determination of chlorophyll-a content proved to be a more reliable and sensitive endpoint, while in dead plant cells, chlorophyll-a begins to decompose rapidly, so the effects of inhibiting algae growth are estimated only based on living cells.

Based on the scientific literature and our results, significant differences can be observed in the sensitivity of different algal and cyanobacterial species to the effects of glyphosate and its formulated herbicides, even within the same taxa [20,70,71,72,113]. Therefore, the significant differences that can be observed in the available toxicity data are not surprising. Differences in the sensitivity of different algal species can presumably be explained by differences in the morphology of different algal cells (e.g., size and shape of cells, surface area to volume ratio, colony formation), the biology of cells (e.g., cell wall permeability, intracellular structure), and the physiology of different species (e.g., growth, nutrient uptake, metabolic activity) [114,115].

During the examination of the phytotoxic effects of glyphosate, the 72 h EC_50_ values determined for *P. subcapitata* (125.2 ± 16.5 and 105.3 ± 17.8 mg/L) far exceed the available literature values (24.7–41 mg/L) [15,73]. The values determined for *D. subspicatus* (132.9 ± 2.3 and 73.8 ± 5.3 mg/L) fit well into the available toxicity range (72.9–166 mg/L) [74,75,76]. The toxicity of GBHs on algae species was investigated in several studies [115,116,117]. The toxicity values determined for Roundup Classic (corrected with glyphosate content) on *P. subcapitata* (72 h EC_50_ values: 5.1 ± 1.3 mg/L) correlated well with some of the published 72 h EC_50_ values (0.7–5.8 mg/L) for Roundup formulations [15,73,118]; however, they remain well below the values published in other studies (15.6–64.7 mg/L) [52,119]. In contrast to the 72 h EC_50_ values demonstrated on the MSDS of the tested formulations for algal test organisms (72 h EC_50_ values = 2.1 mg/L (*P. subcapitata*, Roundup Classic) and 140 mg/L (*D. subspicatus*, Medallon Premium) [97,98]), our toxicity values were significantly higher. During the investigation of the individual and combined toxicity of the components presented in Roundup Classic, the formulating agent POEA proved to be the most toxic component, followed by the formulation, while the toxicity of glyphosate was the lowest on the tested green algae species similar to the results of Tsui and Chu [15]. Similarly, the highest toxicity was observed for the tested surfactant APG compared to the individual toxicity of glyphosate and the combined toxic effects of the formulation on *P. subcapitata* and *D. subspicatus*. However, the highest toxicity was observed after the individual glyphosate exposure on *S. obtusiusculus*. (Table 4, Table 5 and Table 6). The increased toxicity of the formulations in the presence of formulating agents (e.g., POEA) has already been proven in several studies [15,77,120,121]. Based on our results, POEA proved to be more toxic than APG (*p* < 0.001) (Table 4 and Table 6). The determined 72 h EC_50_ values of POEA (2.6 ± 0.7 mg/L and 1.9 ± 0.3 mg/L) roughly correspond to the literature data for *P. subcapitata* (0.2–4.1 mg/L) [15,81,82] (Table 4 and Table 5). The 72 h EC_50_ values determined for APG on *P. subcapitata* (23.0 ± 2.3 mg/L) correspond to the toxicity range determined for long-chain APG compounds (C_12–14_: 11–46 mg/L) [83,84], but far exceed the value (2.7 mg/L) determined by Pavlic et al. [85] for C_10_ carbon chain APG compounds. In contrast, the 72 h EC_50_ values determined for APG compounds with shorter carbon chains (C_8–10_) on *P. subcapitata* proved to be very high (1113–1543 mg/L) [83,84]. The toxicity values determined for *D. subspicatus* (64.3 ± 12.9 mg/L) (Table 6) far exceed the average 72 h EC_50_ values calculated for APG compounds with different chain lengths (C_8–10_: 21 mg/L, C_12–14_: 6 mg/L) [122], but mainly reflect the average value determined by Pavlic et al. [85] (C_10_: 0.32 mg/L). According to the results of several studies, increased toxicity on algae species has been observed with the length of the carbon chain [84,122].

In addition to herbicides that directly inhibit photosynthesis (e.g., atrazine), other active ingredients (e.g., glyphosate) can also affect photosynthesis and respiration processes through their effects on different metabolic pathways [90,91,123]. Based on our results, the pure glyphosate active ingredient did not result in a significant decrease in the photochemical efficiency of the PS II photochemical system compared to the control group up to a concentration of 109 mg/L. On the other hand, in the presence of POEA, a significant decrease was detected in the measured Fv*/Fp values even at a lower concentration of 50.6 mg/L (Figure 1). According to the measured Rfd* values, an increase was observed for both the pure active ingredient and the formulated herbicide preparation at low concentrations (glyphosate: 13.6–27.2 mg/L, Roundup Classic (glyphosate equivalent): 1.5–5.8 mg/L). However, the decrease in the Rfd* values was only observed after the exposure to the herbicide formulation in the tested concentration range (Figure 2).

The adverse effects of glyphosate on the photochemical efficiency of the PS II photochemical system were observed on various green algal species (including *P. subcapitata*) from a concentration of 75 mg/L and several diatom species in the range of 15.3–37.5 mg/L [95]. Moreover, similar to our results, at a lower test concentration (0.02 mg/L), an increase in photosynthetic activity was also observed in the unicellular green algae (*Scenedesmus quadricauda*) [113]. The effects of formulating agents (e.g., POEA, APG) on the photosynthetic activity were investigated on the leaves of higher order plants (*Brassica oleracea*, *Malus domestica*), where significant effects of the tested surfactants were not demonstrated on *M. domestica*. On the other hand, a significant decrease in the photochemical efficiency of the PS II photochemical system was detected on the leaves of *B. oleracea* exposed to POEA [124]. During our measurements, POEA caused a significant decrease in the photochemical efficiency of the PS II photochemical system only in the presence of glyphosate at the highest test concentration (Roundup Classic: 18.9 mg/L POEA equivalent). POEA individually and in the presence of glyphosate induced an increase in Rfd* values at lower test concentrations. Conversely, significantly lower Rfd* values were demonstrated in the higher POEA concentration range compared to the control (Figure 2). After the exposure to Roundup formulations, the phytotoxic effect of glyphosate in the presence of POEA far exceeded the toxicity of the pure active ingredient on the photosynthetic activity of green and blue-green algae species (*M. aeruginosa*, *N. microcarpa var. wrightii*). However, at low concentrations, an increase in photosynthetic activity was also reported [80,90].

The observed stimulatory effects of toxic compounds at lower concentrations can be interpreted as hormesis effects [80,90]. Hormesis is the phenomenon when a xenobiotic has an opposite effect in low and high doses on some infra-individual level property of an organism (biochemical processes, cellular characteristics, histological and organic changes, etc.), or on some characteristic of a population/community [125]. Essentially, hormesis is a biological phenomenon where a harmful compound shows a favorable or stimulating effect in the low concentration range [126]. The phenomenon cannot be characterized by the usual sigmoid (logistic) shaped dose–effect curve [126,127,128]. The explanation of the phenomenon of hormesis is not completely clear, but several background mechanisms can be assumed [127,129]. One of the possible explanations is that the homeostasis of the organism is disturbed by the low concentration of the pollutant and the positive effect appears to compensate the negative effects of the xenobiotics. As a result of the disturbance, the dynamic equilibrium of the body conditions slightly exceeds the normal limits. To compensate for this imbalance, the affected organism mobilizes resources and, in the meantime, achieves a more favorable state than before (e.g., observed higher growth rates by the low concentrations of the tested compounds compared to the control) [127,130]. According to another idea, hormesis results from changes in energy allocation of the organisms [131]. The consequence of the trade-off alterations in life history traits can be indicated by changes in various population parameters (e.g., number of eggs, growth, and behavior) [129,131]. The changes of trade-off caused by pesticides can have an adaptive value, because it helps individuals maintain their fitness [132].

Based on our results, Rfd* proved to be a more sensitive endpoint compared to the Fv*/Fp values characterizing the photochemical efficiency of the PS II photochemical system. During the investigation of the phytotoxic effects, a higher growth rate (3.5 mg/L Roundup Classic) and increased photosynthetic activity were measured at lower test concentrations of the formulation compared to the control groups. At the lower concentrations of the pure active ingredient and POEA, the hormesis effects were only detected during the measurement of the Rfd* values. The observed hormetic effects were also verified by the performed Brain–Cousens hormesis models (*p* < 0.048). Based on the results of algal growth inhibition tests, this stimulating effect was not demonstrated after the individual exposure to the tested components. According to the measured Rfd* values, the hormetic response of *P. subcapitata* was indicated after the individual and combined exposure to the components at the lower concentration ranges. In our study, the observed changes in photosynthetic parameters can presumably be explained primarily by the phenomenon of hormesis, as well as by the change in algal biomass resulting from toxic effects. At low concentrations, the toxic effects do not yet prevail, but on the contrary, the treated algal cells can utilize glyphosate as a source of carbon, phosphorus, and nitrogen [70,90,110,133,134]. Moreover, glyphosate can also trigger pathways for protein and metabolite synthesis [70,133], which can result in increased biomass growth. However, with the increase in concentration, the toxic effects prevail against the excess nutrient content. During the investigation of *Pseudomonas* species, the utilization of octadecyl-bis(2-hydroxyethyl) amine was demonstrated as a carbon and energy source during bacterial growth [81]. Based on certain studies, hormesis can also be interpreted as the response of the plant organism to increased stress [135].

Significant differences were demonstrated in the individual and combined toxicity of the components presented in the tested GBHs. Furthermore, our results support the scientific opinions proposing changes in the official regulations, including the strict regulation of co-formulants, the future development of standards to assess combined effects, and the environmental risks of chemical mixtures [136,137]. Generally, the active ingredients and the co-formulants almost certainly become separated relatively quickly after pesticide treatments. The mobility of the components presented in pesticide formulation highly depends on the physico-chemical properties of the chemical substances (e.g., water solubility, log K_ow_) and the environmental matrices (e.g., pH, level of suspended materials, dissolved oxygen content) [20,21,22,41,138]. The water solubility of glyphosate is 11.6 g/L (25 °C), while degradation half-life (DT_50_) in water varies from a few to 91 days [41]. The water solubility of POEA increases with the increase in oxide–tallow amine ratio [139], while its persistence was demonstrated in soil by several studies [44,45]. The water solubility and biodegradability of APGs depends on the length of the alkyl chain [48]. Various co-formulants affect the solubility and stability of glyphosate, leading to variations in its bioavailability and persistence in the environment [43]. The surfactants applied in GBHs can modify the adsorption capacity of glyphosate, resulting in reduced physical adsorption of glyphosate on the surface of solid–liquid boundary phases (e.g., suspended particles in water samples) [140]. In addition, surfactants form micelles that help glyphosate stay in solution and provide protection against degradation [82,141,142]. In summary, the physico-chemical interactions between glyphosate and the additional ingredients are complex and can significantly influence the overall toxicity of GBHs. Therefore, the ecotoxicological and toxicological evaluation of the various additives is an essential condition for the proper environmental risk assessment of pesticide formulations used in agricultural practice. Currently, manufacturers are only required to indicate the exact chemical name and quantity of the active ingredient(s), synergists, and antidotes on the labels of the products in the EU. Thus, the exact composition of the formulations and information about co-formulants are not public [9,143], resulting in several uncertainties regarding the evaluation of the possible combined toxic effects [144].

However, most of the calculated toxicity values determined for the components presented in glyphosate-based formulations individually and in combination remain below the detected average environmental concentrations in surface waters [27,36], and contamination levels can rise significantly after heavy rains in the watercourses near the treated areas [34,145]. Additionally, the toxicity of glyphosate and POEA can also be significantly influenced by different environmental conditions (e.g., pH, dissolved oxygen content, temperature) [146,147]. Moreover, as a result of global climate change, increased average temperature and the modified intensity of the incident light can significantly influence the phytotoxic effects of glyphosate on phytoplankton communities [91]. Stimulatory effects were indicated at lower concentrations of glyphosate-based Roundup Classic (1.5–5.8 mg glyphosate/L and 0.5–2.2 mg POEA/L), for which concentrations approach or stay much below the measured maximums [27,34,35,37].

## 5. Conclusions

Based on the scientific evidence and our findings, significant differences can be observed in both the individual and combined toxicity of the components contained in the tested GBH formulations. According to our results, the tested co-formulants proved to be the most toxic components. Although the individual toxicity of APG is not as high as for POEA, the toxicity of the formulation is affected by the simultaneous presence of the active ingredient and the co-formulants. Therefore, the revision of GBHs formulated with APG compounds may also be necessary. In addition, significant differences were detected in the sensitivity of the tested algal species, including *D subspicatus* and *S. obtusiusculus* species belonging to the same family (*Scenedesmaceae*). The differences in sensitivity are presumably the result of differences observed in the morphology, cell biology, and physiology of different algal cells. During the evaluation of phytotoxic effects, increased photosynthetic activity was detected on *P. subcapitata* after the exposure to the POEA-formulated GBH and its components in the lower concentration ranges. However, decreased activity was observed after exposure to POEA and the formulation at higher test concentrations. Not only the inhibitory effects but stimulating effects on the growth of algae can adversely affect the aquatic ecosystem and water quality of surface waters. Moreover, the accumulation of phytotoxins can also cause serious environmental effects on aquatic communities.

## Figures and Tables

**Table 2:** Composition and chemical characteristics of the investigated chemical substances.

***Active ingredient (AI)***					
Chemical Name	CAS No.^1^	Concentration of the AI	Physical Appearance	Chemical Structure	
glyphosate isopropylammonium (IPA) salt	38641-94-0	62% (486 g/L glyphosate acid)	water-soluble emulsion	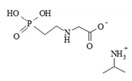	
***Glyphosate-based formulations***					
Product name	AI	Concentration of the AI	Co-formulants	Concentration of the co-formulants	Type of formulation
Roundup Classic	glyphosate IPA salt	41.5% (360 g/L glyphosate acid)	mixture of polyethoxylated tallow amines (POEA)	15.5%	liquid water-soluble concentrate
Medallon Premium	glyphosate diammonium salt(CAS 69254-40-6)	34% (360 g/L glyphosate acid)	alkyl polyglucosides (APG)	10–20%	liquid water-soluble concentrate
***Co-formulants***					
Product name	Co-formulant	Concentration of the co-formulant	Additives	Type of formulation	Chemical structure
Emulson AG GPE 3SS	POEA (CAS 61791-26-2)	100%	–	water-soluble emulsion	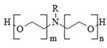
Plantapon LGC	APG (Na-lauryl glucose carboxylate CAS 383178-66-3 + lauryl glucoside CAS 110615-47-9)	28.5–34.0%	water: 66–71.5%	water-soluble emulsion	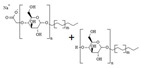

^1^ Chemical Abstracts Service (CAS) Registry Number.

**Table 3:** The main fluorescence parameters and quantities determined by FMM [106].

Fluorescence Parameter	Definition	Interpretation
Fo	observed	Non-variable (original) fluorescence intensity
Fp	observed	Peak fluorescence intensity, maximum fluorescence at a non-saturating light pulse
Fv*	Fp–Fo	Variable fluorescence in terms of Fp
Fv*/Fp	Fv*/Fp	Proxy of quantum efficiency of photosystem II
Fs	observed	Steady-state (terminal) fluorescence
Fd	Fp–Fs	Fluorescence decrease in terms of Fp
Rfd*	Fd/Fs	Fluorescence decrease ratio

**Table 4:** The determined 72 h EC_50_ values for Roundup Classic and its components based on optical density measurements during the ecotoxicological testing on various green algae species.

	72 h EC_50_ Values (mg/L) ^1^			
Algae Species	GLY	Roundup Classic ^2^		POEA
		GLY cont.	POEA cont.	
*Pseudokirchneriella subcapitata*	125.2 ± 16.5	12.2 ± 3.1		2.6 ± 0.7
		5.1 ± 1.3	1.9 ± 0.5	
*Desmodesmus subspicatus*	132.9 ± 2.3	34.0 ± 6.9		4.4 ± 0.4
		14.1 ± 2.9	5.3 ± 1.1	
*Scenedesmus obtusiusculus*	73.1 ± 21.2	65.8 ± 9.0		6.9 ± 1.6
		27.3 ± 3.7	10.2 ± 1.4	

^1^ The combined toxicity of the investigated active ingredient glyphosate (GLY) and formulating agent POEA (mixture of polyethoxylated tallow amines) was investigated in the form of the formulated herbicide preparation. ^2^ The 72 h EC_50_ values for the herbicide formulation corrected with the nominal content of GLY and POEA indicates the concentration of the given component that is present in the formulation causing a 50% effect.

**Table 5:** The determined 72 h EC_50_ values for Roundup Classic and its components based on the chlorophyll-a content during the ecotoxicological testing on green algae species and a cyanobacterium.

	72 h EC_50_ Values (mg/L) ^1^			
Algae Species	GLY	Roundup Classic ^2^		POEA
		GLY cont.	POEA cont.	
*Pseudokirchneriella subcapitata*	105.3 ± 17.8	34.9 ± 3.2		1.9 ± 0.3
		14.5 ± 1.36	5.4 ± 0.5	
*Desmodesmus subspicatus*	73.8 ± 5.3	32.3 ± 9.2		4.9 ± 0.6
		13.4 ± 3.8	5.0 ± 1.4	
*Scenedesmus obtusiusculus*	51.1 ± 2.6	25.4 ± 8.5		4.4 ± 0.9
		10.5 ± 3.5	3.9 ± 0.6	
*Anabaena flos-aquae*	17.4 ± 6.0	n.m. ^3^		n.m.
		n.m.	n.m.	

^1^ The combined toxicity of the investigated active ingredient glyphosate (GLY) and formulating agent POEA (mixture of polyethoxylated tallow amines) was investigated in the form of the formulated herbicide preparation. ^2^ The 72 h EC_50_ values for the herbicide formulation corrected with the nominal content of GLY and POEA indicates the concentration of the given component that is present in the formulation causing a 50% effect. ^3^ not measured.

**Table 6:** The determined 72 h EC_50_ values for Medallon Premium and its components based on the optical density measurements during the ecotoxicological testing on various green algae species.

	72 h EC_50_ Values (mg/L) ^1^			
Algae Species	GLY	Medallon Premium ^2^		APG
		GLY cont.	APG cont.	
*Pseudokirchneriella subcapitata*	125.2 ± 16.5	125.7 ± 13.7		23.0 ± 2.3
		42.7 ± 4.7	18.9 ± 2.1	
*Desmodesmus subspicatus*	132.9 ± 2.3	720.9 ± 96.6		64.3 ± 12.9
		245.1 ± 32.8	108.1 ± 14.5	
*Scenedesmus obtusiusculus*	73.1 ± 21.2	687.5 ± 171.9		137.9 ± 19.1
		233.8 ± 58.4	103.1 ± 25.8	

^1^ The combined toxicity of the investigated active ingredient glyphosate (GLY) and formulating agent APG (alkyl polyglucosides) was investigated in the form of the formulated herbicide preparation. ^2^ The 72 h EC_50_ values for the herbicide formulation corrected with the nominal content of GLY and APG indicates the concentration of the given component that is present in the formulation causing a 50% effect.

## Data Availability

The data presented in this study are available on request from the corresponding author. The data are not publicly available due to privacy reasons.
